# Antimicrobial Resistance Distribution Differs Among Methicillin Resistant *Staphylococcus aureus* Sequence Type (ST) 5 Isolates From Health Care and Agricultural Sources

**DOI:** 10.3389/fmicb.2018.02102

**Published:** 2018-09-11

**Authors:** Samantha J. Hau, Jisun S. Haan, Peter R. Davies, Timothy Frana, Tracy L. Nicholson

**Affiliations:** ^1^Department of Veterinary Diagnostic and Production Animal Medicine, College of Veterinary Medicine, Iowa State University, Ames, IA, United States; ^2^Enterics Unit-Infectious Disease Lab, Public Health Laboratory Division, Minnesota Department of Health, Saint Paul, MN, United States; ^3^Department of Veterinary Population Medicine, College of Veterinary Medicine, University of Minnesota, Saint Paul, MN, United States; ^4^National Animal Disease Center, Agricultural Research Service, United States Department of Agriculture, Ames, IA, United States

**Keywords:** LA-MRSA, *Staphylococcus aureus*, antimicrobial resistance, swine, mobile genetic elements, agriculture

## Abstract

Antimicrobial resistance (AMR) is an expanding public health concern and methicillin resistant *Staphylococcus aureus* (MRSA) is a notable example. Since the discovery of livestock associated MRSA (LA-MRSA), public health concerns have arisen surrounding the potential of LA-MRSA isolates to serve as a reservoir for AMR determinants. In this study, we compare swine associated LA-MRSA ST5 and human clinical MRSA ST5 isolates for phenotypic antimicrobial susceptibilities determined via broth microdilution and genotypic determinants of AMR using whole genome sequencing and comparative genomic analysis to identify AMR elements. Swine associated LA-MRSA ST5 isolates exhibited phenotypic resistance to fewer antibiotics than clinical MRSA ST5 isolates from humans with no swine contact. Distinct genomic AMR elements were harbored by each subgroup, with little overlap in shared AMR genes between swine associated LA-MRSA ST5 and clinical MRSA ST5 isolates. Our results demonstrate that phenotypic antimicrobial susceptibilities and genotypic determinants of AMR among swine associated LA-MRSA ST5 and clinical MRSA ST5 isolates are separate and distinct.

## Introduction

Treatment of *Staphylococcus aureus* infections is complicated by the acquisition of mobile genetic elements (MGEs) encoding antimicrobial resistance (AMR). Most notable of these is the SCC*mec* element harboring *mecA* (less commonly *mecB* or *mecC*) which encodes resistance to methicillin (Jevons, 1961). Methicillin resistant *S. aureus* (MRSA) are classified epidemiologically based on their putative source into hospital acquired (HA-MRSA), community acquired (CA-MRSA), and livestock associated (LA-MRSA); however, these definitions have become blurred with some MRSA lineages being identified in multiple settings.

Methicillin resistant *S. aureus* infections were only attributed to or isolated from hospital settings until the 1990s, when CA-MRSA isolates were detected in community members with no risk factors for HA-MRSA (Pantosti, 2012). Although CA-MRSA isolates are considered more virulent than HA-MRSA isolates, HA-MRSA isolates typically harbor a greater number of AMR determinants (Klevens et al., 2007; Pantosti, 2012). LA-MRSA was first reported in swine in 2005 and raised concerns that swine and other livestock may serve as reservoirs for MRSA isolates that can transmit to humans (Voss et al., 2005). While LA-MRSA are less able to colonize and cause disease in humans than HA- and CA-MRSA isolates (Cuny et al., 2009; Uhlemann et al., 2012; Walter et al., 2016), they often harbor multiple AMR genes and can be a source for genes encoding uncommon AMR determinants, such as the multidrug resistance gene *cfr* and the lincosamide, pleuromutilin, and streptogramin A resistance genes *vga*(C) and *vga*(E) (Kadlec et al., 2012; Li et al., 2015; Aires-de-Sousa, 2016).

Multi-locus sequence typing (MLST) has been employed to group *S. aureus* isolates into sequence types (STs) that share alleles at seven MLST loci. The distribution of STs in both human and animal populations, including swine, varies regionally. In Europe, ST398 is the predominant swine associated LA-MRSA lineage and in Asia, ST9 isolates dominate (Andreoletti et al., 2009; Wagenaar et al., 2009). In contrast, swine herds in North America harbor a mixed population of LA-MRSA isolates containing ST398, ST9, and ST5 (Weese et al., 2010; Frana et al., 2013). While ST9 and ST398 MRSA isolates are considered livestock adapted and are uncommon causes of human infections in most settings (Pantosti, 2012; Becker et al., 2015), ST5 isolates compose a globally disseminated and highly successful lineage with both CA- and HA-MRSA clones reaching pandemic levels (Monecke et al., 2011). The success of the ST5 lineage in and out of a hospital setting is attributed to the capacity of these isolate to acquire MGEs containing genes encoding virulence factors and AMR (Monecke et al., 2011).

Antimicrobial resistance is a significant public health concern due to the economic and societal cost associated with increased morbidity, mortality, and treatment costs (Maragakis et al., 2008). Both ST398 and ST9 LA-MRSA isolates can harbor diverse resistance elements (Kadlec et al., 2012; Yan et al., 2016), raising concerns over the potential for LA-MRSA isolates to disseminate AMR beyond the agricultural setting. In this report, we evaluate the prevalence and diversity of AMR phenotypes and genetic determinants conferring AMR between LA-MRSA ST5 isolates from a variety of swine associated sources and clinical MRSA ST5 isolates from humans with no swine contact to assess isolates for shared resistance elements or phenotypic resistance patterns.

## Materials and Methods

### Isolate Acquisition

Eighty-two swine associated LA-MRSA ST5 isolates were obtained from Iowa State University and the University of Minnesota. Sources for the isolates included nasal swabs from healthy pigs (*n* = 38) and environmental samples (*n* = 26) obtained from eight swine farms, as well as nasal swabs from healthy humans not exhibiting any signs of MRSA related disease with short-term (*n* = 9) and long-term (*n* = 9) swine contact (Frana et al., 2013). Seventy-one clinical MRSA isolates from humans with no known livestock contact were obtained from healthcare settings at the University of California San Francisco (*n* = 7) and the University of California Irvine (*n* = 64; Hudson et al., 2013). All isolates were determined to be *mecA* positive and were MLST, SCC*mec*, and *spa* typed prior to acquisition (**Supplementary Table S1**). All isolates were either obtained from samples collected as part of previous studies (Frana et al., 2013; Hudson et al., 2013) or were obtained from samples submitted as part of field case investigations and did not require Institutional Animal Care and Use Committee (IACUC) approval.

### DNA Sequencing

Genomic DNA was extracted and sequenced as described previously (Hau et al., 2017a,b,c,d,e,f). Briefly, genomic DNA was extracted from isolates grown in Trypticase Soy Broth (BD Biosciences, Sparks, MD, United States) using the High Pure PCR Template Preparation Kit (Roche Applied Science, Indianapolis, IN, United States). The Nextera XT DNA sample preparation and index kit (Illumina, San Diego, CA, United States) was used to generate indexed libraries that were pooled and sequenced on an Illumina MiSeq instrument using the MiSeq v2 500 Cycle reagent kit (Illumina, San Diego, CA, United States). Sequence reads were assembled using MIRA v. 4.0.2^1^ (Chevreux et al., 1999).

### Genomic AMR Analysis

ResFinder 2.1 from the Center for Genomic Epidemiology^2^ and the Comprehensive Antibiotic Resistance Database (CARD)^3^ were employed for AMR identification. Draft genomes were submitted to ResFinder 2.1 using a threshold ID of 70% and a minimum length of 60% and CARD using the criteria “default – perfect and strict hits only.”

Antimicrobial resistance genetic elements were analyzed using Geneious 9.0.5 (Biomatters Ltd., Auckland, New Zealand). Multiple sequence alignments were used to compare sequence identity of genes and plasmids from different isolates. AMR elements found on transposons were evaluated for location of integration. To confirm gene results from ResFinder and evaluate MGE containing resistance determinants, sequence information was submitted to the National Center for Biotechnology Information Basic Local Alignment Search Tool (BLAST) for comparison with the sequence database^4^. Geneious 9.0.5 (Biomatters Ltd., Auckland, New Zealand) was used for image generation. AMR genetic elements harbored by each isolate were utilized to generate a presence/absence binary table, which was converted into a distance matrix and clustered by means of average hierarchical clustering based on Euclidean Distance using TM4 (Saeed et al., 2003).

### Phenotypic AMR Analysis

Phenotypic antibiotic resistance was determined using the broth microdilution method by Iowa State University following standard operating procedures. Minimum inhibitory concentrations (MICs) were determined for each isolate using the Trek BOPO6F plate (Thermo Fisher Scientific Inc., Oakwood Village, OH, United States) and the Trek GPALL1F plate (Thermo Fisher Scientific Inc., Oakwood Village, OH, United States) with *S. aureus* 29213 (ATCC, Manassas, VA, United States) serving as the control strain. MICs were evaluated in accordance with Clinical Laboratory Standards Institute (CLSI) recommendations based on Vet01-A4 and M100 25th edition standards to give resistance interpretations for 29 antibiotics in 13 antibiotic classes (**Table 1**). AMR index, defined as the proportion of antibiotics tested to which an isolate exhibited phenotypic resistance, was determined for each isolate using the results of the microbroth dilution analysis.

**Table 1:** Antibiotic resistance prevalence for screened antibiotics in LA-MRSA ST5 isolates and clinical MRSA ST5 isolates.

Antibiotic class	Antibiotic	Swine associated LA-MRSA ST5^a^	Clinical MRSA ST5	Statistics^b^
Beta-lactam	Penicillin	82/82 (100%)	71/71 (100%)	NS, *P* = 1.0
	Ampicillin	82/82 (100%)	71/71 (100%)	NS, *P* = 1.0
	Oxacillin	78/82 (95.1%)	71/71 (100%)	NS, *P* = 0.1240
	Cefoxitin	74/82 (90.2%)	71/71 (100%)	*P* = 0.0075
	Ceftiofur	73/82 (89.0%)	67/71 (94.4%)	NS, *P* = 0.2628
	Ceftriaxone	49/82 (59.8%)	60/71 (84.5%)	*P* = 0.0011
Aminoglycoside	Gentamicin	22/82 (26.8%)	12/71 (16.9%)	NS, *P* = 0.1735
	Neomycin	69/82 (84.1%)	67/71 (94.4%)	NS, *P* = 0.0689
	Streptomycin	1/82 (1.2%)	1/71 (1.4%)	NS, *P* = 1.0
Tetracycline	Chlortetracycline	65/82 (79.3%)	0/71 (100%)	*P* < 0.0001
	Oxytetracycline	65/82 (79.3%)	0/71 (100%)	*P* < 0.0001
	Tetracycline	65/82 (79.3%)	0/71 (100%)	*P* < 0.0001
Phenicols	Chloramphenicol	9/82 (11.0%)	1/71 (1.4%)	*P* = 0.0206
	Florfenicol	30/82 (36.6%)	54/71 (76.1%)	*P* < 0.0001
Macrolide	Erythromycin	36/82 (43.9%)	69/71 (97.2%)	*P* < 0.0001
	Tilmicosin	36/82 (43.9%)	55/71 (75.3%)	*P* < 0.0001
Sulfonamides	Sulfadimethoxine	0/82 (0%)	35/71 (49.3%)	*P* < 0.0001
	Trimethoprim + sulfamethoxazole	0/82 (0%)	0/71 (0%)	NS, *P* = 1.0
	Trimethoprim	0/82 (0%)	0/71 (0%)	NS, *P* = 1.0
Fluoroquinolones	Ciprofloxacin	19/82 (23.2%)	69/71 (97.2%)	*P* < 0.0001
	Enrofloxacin	25/82 (30.5%)	69/71 (97.2%)	*P* < 0.0001
	Levofloxacin	15/82 (18.3%)	69/71 (97.2%)	*P* < 0.0001
	Moxifloxacin	18/82 (22.0%)	69/71 (97.2%)	*P* < 0.0001
Nitrofuran	Nitrofurantoin	0/82 (0%)	0/71 (0%)	NS, *P* = 1.0
Lincosamide	Clindamycin	39/82 (47.6%)	55/71 (77.5%)	*P* = 0.0002
Lipopeptide	Daptomycin	0/82 (0%)	0/71 (0%)	NS, *P* = 1.0
Pleuromutilin	Tiamulin	7/82 (8.5%)	1/71 (1.4%)	NS, *P* = 0.0654
Glycopeptide	Vancomycin	0/82 (0%)	0/71 (0%)	NS, *P* = 1.0
Oxazolidinone	Linezolid	0/82 (0%)	0/71 (0%)	NS, *P* = 1.0

*^a^Number resistant out of total isolates tested (percent resistant). ^b^Statistical significance designated at P < 0.05; NS, not significant.*

### Statistical Analysis

Statistical analysis was completed using GraphPad Prism 7.01 (GraphPad Software, Inc., La Jolla, CA, United States). Phenotypic AMR comparisons were completed with contingency analysis. Comparisons of AMR index and resistance gene numbers were completed using Mann–Whitney tests. Results were considered significant using a *P*-value cutoff of *P* < 0.05.

## Results

### Phenotypic AMR Distribution

**FIGURE 1:**
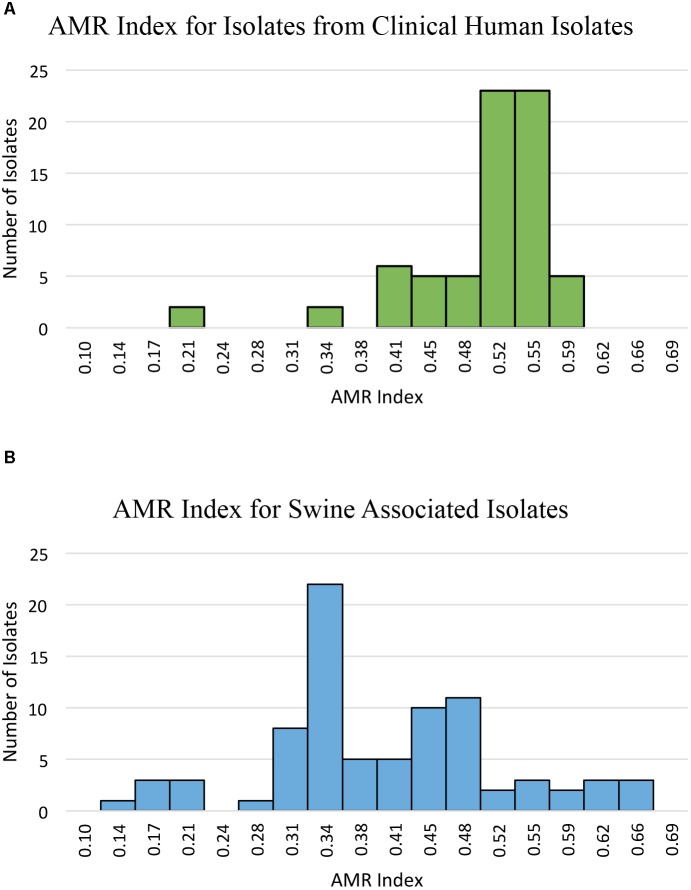
AMR index of isolates from clinical MRSA ST5 and swine associated LA-MRSA ST5. AMR index is defined as the proportion of the tested antibiotics to which an isolate is phenotypically resistant. **(A)** The AMR indexes determined for clinical isolates show a range of 0.21–0.59 with an average AMR index of 0.50. **(B)** The AMR indexes determined for swine associated LA-MRSA ST5 isolates. Swine associated isolates had AMR indexes with a wider range (0.14–0.66) and higher maximum AMR index; however, the average AMR index was 0.40, which was significantly less than that of clinical isolates (*P* < 0.0001).

Phenotypic resistance was determined and prevalence was compared between swine-associated LA-MRSA ST5 isolates and clinical MRSA ST5 isolates (**Table 1**). Swine associated isolates had AMR indices ranging from 0.14–0.66, while clinical isolates had AMR indices ranging from 0.21–0.59 (**Figure 1**). Clinical MRSA ST5 isolates had significantly higher AMR index (median = 0.52) than LA-MRSA ST5 isolates (median = 0.38; *P* < 0.0001), which equated to phenotypic resistance to an average of 14.6 and 11.7 (median 15 and 11) antibiotics, respectively (**Figure 1**). These data indicate that AMR was generally less extensive among swine associated LA-MRSA ST5 isolates than clinical MRSA ST5.

Isolates were screened for resistance to vancomycin and linezolid, antibiotics of choice for MRSA treatment in a hospital setting. Neither swine associated nor clinical MRSA ST5 isolates displayed phenotypic resistance to vancomycin or linezolid (**Table 1**). Genetic determinants conferring vancomycin resistance and the multidrug resistance gene *cfr* were absent from the genomes of all isolates.

### Fluoroquinolone Resistance

Phenotypic resistance to fluoroquinolone antibiotics was significantly more prevalent among clinical MRSA ST5 isolates than swine associated LA-MRSA ST5 isolates (*P* < 0.0001; **Table 1**). Of the fluoroquinolone resistant LA-MRSA ST5 isolates, 13/25 (52.0%) were obtained from a single farm and 4/25 (16%) isolates were from humans after short-term swine contact on that farm. The remaining fluoroquinolone resistant isolates (8/25, 32.0%) were distributed on two other farms (*n* = 4), from humans contacting swine on those farms (*n* = 2), or from humans with long term swine contact (*n* = 2).

Isolates were screened for chromosomal mutations and genes conferring fluoroquinolone resistance. In isolates obtained from humans with no swine contact, fluoroquinolone resistance was associated with mutations in *gyrA*, *parC*, and/or *parE*, while resistance in swine associated isolates was primarily associated with mutations in both *gyrA* and *parC* (**Table 2**). Eight isolates from humans with no swine contact had no identified resistance determinants. This may be a result of gaps in the draft genome, novel mutations, or novel genes conferring fluoroquinolone resistance. Eight swine associated isolates also lacked a mutation or AMR gene conferring fluoroquinolone resistance. One of these isolates was associated with the farm harboring isolates with *parC* and *gyrA* mutations, indicating the mutations may not be present due to gaps in the draft genome sequence. Of the remaining seven isolates, six exhibited limited phenotypic resistance, being resistant only to enrofloxacin but susceptible to ciprofloxacin, levofloxacin, and moxifloxacin. These isolates also exhibited a lower enrofloxacin MIC (2.0 μg/mL) when compared to isolates with *gyrA*, *parC*, or *parE* mutations (MIC of > 2.0 μg/mL). Other isolates from the same farms showed a range of MICs from < 0.12–1.0 μg/mL with 21/33 (63.6%) of the non-resistant isolates from these farms of “intermediate” susceptibility.

**Table 2:** AMR genes identified.

Resistance genes	Swine associated	Clinical MRSA ST5
	LA-MRSA ST5	
**Fluoroquinolone resistance**		
*gyrA* mutation	0/82 (0%)	10/71 (14.1%)
*parC* mutation	1/82 (1.2%)	11/71 (15.5%)
*parE* mutation	0/82 (0%)	3/71 (4.2%)
*gyrA + parC* mutation	16/82 (19.5%)	33/71 (46.5%)
*gyrA + parC + parE* mutation	0/82 (0%)	4/71 (5.6%)
Unknown	8/82 (9.8%)	8/71 (11.3%)
**Tetracycline resistance**		
*tet*(L)	62/82 (75.6%)	0/71 (0%)
*tet*(T)	61/82 (74.4%)	0/71 (0%)
Unknown	3/82 (3.7%)	0/71 (0%)
**Macrolide, lincosamide, streptogramin resistance**		
*erm*(A)	0/82 (0%)	65/71 (91.5%)
*erm*(C)	36/82 (43.9%)	0/71 (0%)
*vga*(A)	78/82 (95.1%)	0/71 (0%)
*vga*(E)	2/82 (2.4%)	0/71 (0%)
*mph*(C)	0/82 (0%)	20/71 (28.2%)
*msr*(A)	0/82 (0%)	20/71 (28.2%)
*lnu*(B)	1/82 (1.2%)	0/71 (0%)
*lnu*(A)	0/82 (0%)	2/71 (2.8%)
Unknown	2/82 (2.4%)	4/71 (5.6%)
**Aminoglycoside Resistance**		
*aadD*	71/82 (86.6%)	62/71 (87.3%)
*aadE*	1/82 (1.2%)	0/71 (0%)
*aph(2″)-Ih*	2/82 (2.4%)	3/71 (4.2%)
*aac(6′)-aph(2″)*	14/82 (17.1%)	9/71 (12.7%)
*spc*	0/82 (0%)	61/71 (85.9%)
*str*	1/82	0/71 (0%)
*aph(3′)-III*	0/82 (0%)	15/71 (21.1%)
*ant(6)-Ia*	0/82 (0%)	15/71 (21.1%)
Unknown	3/82 (3.7%)	0/71 (0%)

### Tetracycline Resistance

Tetracycline resistance was seen exclusively in swine associated LA-MRSA ST5 isolates (**Table 1**). Resistance was present on all but one farm (7/8, 87.5%). Genetic screening for AMR genes indicated swine associated LA-MRSA ST5 isolates harbored zero to two tetracycline resistance genes (**Supplementary Table S1**). The tetracycline resistance genes identified in LA-MRSA ST5 isolates were *tet*(T) (61/82, 74.4%) and *tet*(L) (62/82, 75.6%) (**Table 2**). The majority (61/65, 93.8%) of isolates phenotypically resistant to tetracycline harbored both *tet*(T), a ribosomal modification gene, and *tet*(L), a gene encoding antibiotic efflux. Sequence examination revealed tetracycline resistance genes were located on a plasmid also harboring the aminoglycoside resistance gene *aadD* (**Supplementary Figure S1**). Consistent with the lack of phenotypic resistance, no tetracycline resistance genes were identified in the genomes of clinical MRSA ST5 isolates (0/73, 0%; **Table 2**).

### Macrolide, Lincosamide, and Streptogramin Resistance

Phenotypic resistance to macrolide (*P* < 0.0001) and lincosamide (*P* = 0.0002) antibiotics was significantly higher in clinical MRSA ST5 isolates than LA-MRSA ST5 isolates (**Table 1**). Genomic screening for determinants conferring resistance to MLS antibiotics revealed differences in resistance genes between the subsets of isolates. The majority of clinical MRSA ST5 isolates harbored the *erm*(A) gene (65/71, 91.5%; **Table 2**). These isolates presented with two distinct phenotypes: 14/65 (21.5%) were resistant to erythromycin and susceptible to tilmicosin and clindamycin and 51/65 (78.5%) isolates were resistant to erythromycin, tilmicosin, and clindamycin. Genome sequence analysis revealed the *erm*(A) gene was contained within the type II SCC*mec* element in 65/69 (94.2%) of the erythromycin resistant isolates. The remaining four isolates also harbored a type II SCC*mec* element, indicating the *erm*(A) gene may be present but missing from the draft sequence. The remaining isolates (2/71, 8.5%) were susceptible to tested MLS antibiotics and harbored a type IV SCC*mec* element, which does not harbor *erm*(A).

Clinical isolates also harbored several MLS resistance genes other than *erm*(A) (**Table 2**). Two isolates harbored *lnu*(A), a gene that functions to inactivate lincosamide antibiotics. There were also 20 isolates (28.2%) that harbored *mph*(C), a gene involved in macrolide antibiotic inactivation, and *msr*(A), a streptogramin and macrolide efflux pump (**Table 2**). These genes [*lnu*(A), *mph*(C), and *msr*(A)] were found in isolates harboring *erm*(A) or isolates suspected to have *erm*(A) based on their SCC*mec* type (**Supplementary Table S1**). Due to the resistance profile of *erm*(A) harboring isolates, the contribution of *lnu*(A), *mph*(C), and *msr*(A) to macrolide resistance was not able to be determined.

Swine associated isolates with phenotypic resistance to macrolide and lincosamide antibiotics harbored *erm*(C) (36/82, 43.9%; **Table 2**). These isolates displayed one phenotype: resistance to erythromycin, tilmicosin, and clindamycin. Sequence analysis determined that *erm*(C) was plasmid mediated. Two different plasmids were identified with the majority of *erm*(C) positive isolates (35/39, 89.7%) containing a 2.4 kbp plasmid encoding only *erm*(C) and a maintenance and replication protein (**Supplementary Figure S2**). There was a single swine associated isolate harboring the lincosamide resistance gene *lnu*(B) and it expressed phenotypic lincosamide resistance.

The majority of swine associated LA-MRSA strains harbored an AMR gene conferring resistance to streptogramin A antibiotics. The most common streptogramin A resistance gene was *vga*(A) gene (78/82, 95.1%), which functions as an efflux pump with activity toward streptogramin A antibiotics and lincosamide antibiotics. Two isolates harbored *vga*(E) along with *vga*(A). There was no correlation between phenotypic lincosamide resistance and the presence of *vga*(A) or *vga*(E). No streptogramin resistance genes were identified in clinical MRSA ST5 isolates.

### Aminoglycoside Resistance

Phenotypic aminoglycoside resistance (gentamicin, neomycin, or streptomycin) was not significantly different in swine associated and clinical MRSA ST5 isolates (**Table 1**). Neomycin resistance was widely distributed in LA-MRSA ST5 isolates and was present in isolates from all farms sampled (8/8, 100%). Phenotypic gentamicin and streptomycin resistance was less widely distributed in LA-MRSA ST5 isolates, with all gentamicin resistant isolates obtained from a single farm. Streptomycin resistance was identified in one swine associated isolate.

Genetic determinants conferring aminoglycoside resistance were more prevalent in clinical MRSA ST5 isolates (range 0–5, average 2.3, median 2) than isolates from swine associated sources (range 0–3, average 1.1, median 1; *p* < 0.0001; **Table 2**). The aminoglycoside resistance genes unique to clinical MRSA isolates were *spc*, *aph(3′)-III*, and *ant(6)-Ia*. Isolates from swine associated sources uniquely harbored *aadE* and *str* (**Table 2**). Three resistance genes were shared between the two groups of isolates: *aadD*, *aph(2″)-Ih*, and *aac(6′)-aph(2″)* (**Table 2**).

Neomycin resistance was widely distributed in both swine associated LA-MRSA ST5 isolates (69/82, 84.1%) and clinical MRSA ST5 isolates (67/71, 94.4%) and correlated with the presence of *aadD* in both subsets of isolates (**Supplementary Table S1**). A strong association (100% in clinical isolates and 72.7% in swine associated isolates) between phenotypic resistance to gentamicin and the aminoglycoside resistance gene *aac(6′)-aph(2″)* or *aph(2″)-Ih* was also observed (**Supplementary Table S1**). Further genetic investigation indicated that the gene identified as *aph(2″)-Ih* is likely a truncated version of *aac(6′)-aph(2″)* found at the end of the contig. Finally, a single swine associated isolate carried the resistance gene *str*. This isolate was also the only swine associated ST5 displaying phenotypic resistance to streptomycin. The clinical isolate exhibiting phenotypic resistance to streptomycin did not harbor an identified aminoglycoside resistance gene.

Sequence analysis was used to determine the precise location of the shared aminoglycoside resistance genes to detect evidence of potential transfer between the two subsets of isolates. The location of *aadD* varied among LA-MRSA ST5 isolates. The majority of isolates (62/71, 87.3%) harbored *aadD* on a multidrug resistance plasmid that also harbored the tetracycline resistance genes *tet*(L) and *tet*(T) (**Supplementary Figure S1**). In the remaining nine LA-MRSA isolates, *aadD* was harbored on different plasmids. Some of these plasmids contained other AMR genes, such as the beta-lactamase, *blaZ*. In the majority (59/62, 95.2%) of isolates from humans with no swine contact, *aadD* was identified within plasmid sequence that also contained a bleomycin resistance gene (**Supplementary Figure S3**). Of the remaining isolates, two harbored *aadD* on a contig encoding a bacitracin ABC transporter permease; however, whether this contig was plasmid or chromosomal sequence could not be determined. Evaluation of the location of *aac(6′)-aph(2″)* indicated that all isolates harbored a similar insertion sequence containing the gene (**Supplementary Figure S4**). BLAST results indicated the insertion sequence was present in several plasmids, none of which were common between the subsets of isolates.

### Phenicol Resistance

Phenotypic resistance to phenicol antibiotics differed between swine associated and clinical MRSA ST5 isolates (**Table 1**). Significantly more swine associated isolates exhibited phenotypic resistance to chloramphenicol (*P* = 0.02); while resistance to florfenicol was more prevalent in isolates from humans with no swine contact (*P* < 0.0001).

Genotypically, all swine associated isolates exhibiting phenotypic chloramphenicol resistance (9/9, 100%) harbored the phenicol resistance gene *fexA* (**Table 2**). These isolates were also phenotypically resistant to florfenicol (**Supplementary Table S1**). The presence of *fexA* and chloramphenicol resistance were clustered in swine associated isolates and all *fexA* containing isolates were obtained from a single farm. No genetic determinants conferring chloramphenicol resistance were identified in the genome of the clinical isolate that exhibited phenotypic resistance to chloramphenicol. All florfenicol resistant clinical isolates and the majority of florfenicol resistant swine associated isolates (21/30, 70%) did not harbor any recognized genes encoding florfenicol resistance.

### Genotypic Resistance Profile

**FIGURE 2:**
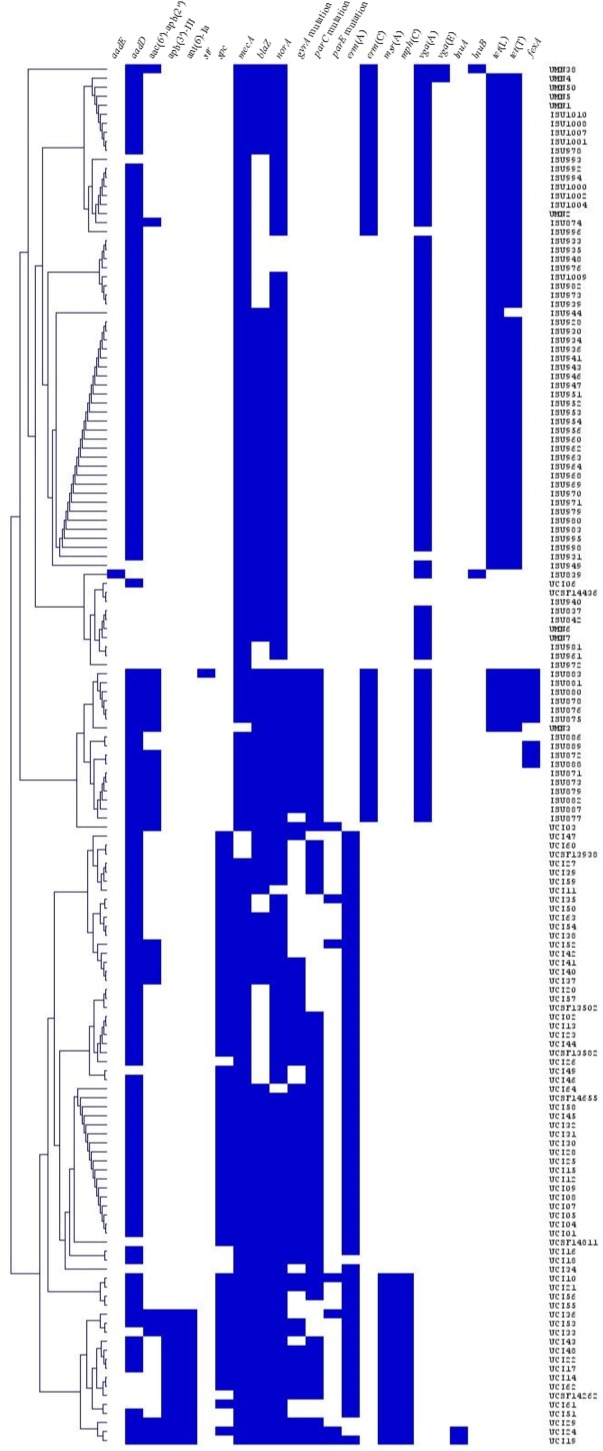
Hierarchical cluster dendrogram displaying the relatedness of the MRSA ST5 isolates based on the presence or absence of AMR genetic elements. Isolates are grouped by average hierarchical clustering based on Euclidean Distance. AMR genetic elements are listed at the top, isolate names are listed to the right, and presence (blue) or absence (white) of genes is indicated.

The AMR genes harbored by each isolate were utilized to generate a hierarchical cluster dendrogram representing isolate similarity based on presence or absence of AMR genetic elements (**Figure 2**). Two clades were generated, one containing 69/71 (97%) of the clinical MRSA ST5 isolates and the other harboring all swine associated MRSA ST5 isolates and two clinical MRSA ST5 isolates (UCI06 and UCSF14436). The two clinical isolates within the swine associated clade were found to harbor only three AMR genes (*mecA*, *blaZ*, and *norA*) and the absence of additional resistance genes contributed to their presence within the swine associated clade. The clade separation of the clinical and swine associated MRSA ST5 isolates indicates distinct differences in the resistance genes harbored by the subsets of isolates.

## Discussion

While previous studies have reported AMR prevalence among LA-MRSA ST398 and ST9 isolates (Argudin et al., 2010, 2011; Nemeghaire et al., 2014; Moon et al., 2015; Mutters et al., 2016; Li et al., 2017), little to no information exists regarding AMR prevalence among LA-MRSA ST5 isolates. We found clinical ST5 isolates exhibited a higher AMR index than ST5 isolates obtained from swine associated sources. In contrast to this general trend, 13 (15.9%) LA-MRSA ST5 isolates displayed resistance to 15–19 of the antibiotics tested (AMR index of 0.52–0.66; **Figure 1**). Nine of these isolates were from a single farm and one was obtained from a human with short-term swine contact on that farm. Multidrug resistance patterns similar to that observed in this farm have also been reported in swine associated ST398 LA-MRSA isolates (Kadlec et al., 2009; Argudin et al., 2010; Morcillo et al., 2015; Mutters et al., 2016). Additionally, our findings contrast with that seen in a recent report by You et al. (2018), which found a higher prevalence and diversity of AMR determinants in *S. aureus* from slaughterhouse workers as compared to *S. aureus* isolates obtained from community members. However, the genetic characteristics and AMR profile of the isolates from slaughterhouse workers indicate these isolates are more consistent with CA-MRSA than LA-MRSA, likely contributing to the observed differences AMR prevalence as compared to our study (You et al., 2018).

Antimicrobial resistance distribution among swine associated MRSA ST5 isolates predominantly reflected patterns consistent with antimicrobial use in the swine industry. Tetracycline resistance was found in 79.3% of the LA-MRSA ST5 isolates and on seven of the eight farms sampled. Resistance to tetracyclines has been previously reported in LA-MRSA ST398 isolates, where phenotypic resistance has approached 100% of isolates evaluated (Kadlec et al., 2009; Argudin et al., 2011; Zarfel et al., 2013; Peeters et al., 2015; Mutters et al., 2016). This has been attributed to the long term use of chlortetracycline and oxytetracycline antibiotics in the swine industry. Similarly, the much lower prevalence of fluoroquinolone resistance in LA-MRSA ST5 is unsurprising given the relatively recent approval (2008) of fluoroquinolones for swine in the United States and the ban on extra label use of fluoroquinolone antibiotics in food animals under the Animal Medicinal Drug Use Clarification Act (AMDUCA) (Food and Drug Administration, 2012). While the prevalence of chloramphenicol resistance in LA-MRSA ST5 isolates was similar to that reported for LA-MRSA ST398 (Argudin et al., 2011; Peeters et al., 2015), we noted higher chloramphenicol resistance in swine associated LA-MRSA ST5 isolates as compared to clinical ST5 isolates, though this antibiotic is banned under AMDUCA. The presence of chloramphenicol resistance among both LA-MRSA ST398 and ST5 isolates may reflect selection related to the use of florfenicol in the swine industry.

Evaluation of the draft genome sequences revealed the AMR genes identified in the MRSA ST5 isolates were harbored on MGE for all antibiotics except for fluoroquinolones. The AMR genes were found on plasmids [such as *tet(*L*)*, tet(T), *aadD*, *erm*(C), *msr*(A), and *mph*(C)], transposons [such as *aac(6′)-aph(2″)*, *lnu*(B), *aadE*, and *fexA*], and within the SCC*mec* element [*erm*(A) and *ant(9)-Ia*]. The presence of AMR genes detected in this study on MGE underlies the potential for the transfer of AMR genes among bacteria. *S. aureus* ST5 isolates are highly susceptible to transfer of MGE (Monecke et al., 2011), which may facilitate transmission of uncommon AMR determinants to and from LA-MRSA ST5 isolates.

Notable differences in genetic determinants underlying AMR were identified in LA-MRSA ST5 isolates compared to clinical MRSA ST5 isolates (**Figure 2** and **Table 1**). MLS resistance was mediated by the *erm*(A) gene in clinical human isolates and the *erm*(C) gene in swine associated isolates. While a genetic determinant conferring resistance to phenicol class antibiotics was unidentified in the majority of florfenicol resistant isolates, *fexA* was harbored by a portion of LA-MRSA ST5 isolates expressing phenicol resistance (9/30, 30%) but absent from all phenicol resistant clinical MRSA ST5 isolates. There were two shared AMR genes between LA-MRSA ST5 isolates and clinical MRSA ST5 isolates: *aadD* and *aac(6′)-aph(2″)*. For these genes, although sequence analysis showed sequence identity for both genes to be high (>90% across all isolates), the genes were harbored on different plasmids in the two subsets of isolates. Ultimately, this indicates it is unlikely the presence of these genes in clinical MRSA ST5 isolates was associated with transfer from LA-MRSA ST5 isolates carrying the genes. Additionally, when isolates were phylogenetically grouped by the presence or absence of AMR genes, there was a distinct separation between clinical MRSA ST5 isolates and LA-MRSA ST5 isolates. Two clinical isolates were found within the swine associated clade; however, these isolates were clustered with LA-MRSA due to the absence of AMR genes rather than the presence of AMR genes shared with LA-MRSA ST5 isolates. Similarly, separate and distinct sets of AMR genes harbored by human isolates compared to livestock associated isolates were previously reported for *Salmonella* Typhmurium DT104 and in comparisons between LA-MRSA and HA-MRSA isolates in the same location (Mather et al., 2013; Mutters et al., 2016).

Interestingly, LA-MRSA ST5 isolates harbored a different complement of AMR genes than those previously identified in LA-MRSA ST398 isolates. For example, tetracycline modification genes and tetracycline efflux genes were widespread in the LA-MRSA ST5 isolates tested here and in LA-MRSA ST398 (Argudin et al., 2011; Price et al., 2012; Peeters et al., 2015; Mutters et al., 2016). While both STs can harbor *tet*(L), tetracycline resistance genes [*tet*(M) and *tet*(K)] previously identified in LA-MRSA ST398 were not found in our LA-MRSA ST5 isolates. Similarly, MLS resistance genes in LA-MRSA ST398 have been more diverse than those we identified in LA-MRSA ST5 (Argudin et al., 2011; Peeters et al., 2015; Mutters et al., 2016). The prevalence of *erm*(C) ranged from 16–40% in previous reports of LA-MRSA ST398 isolates (Argudin et al., 2011; Peeters et al., 2015; Mutters et al., 2016), in which *erm*(A) (0–34% of isolates) and *erm*(B) (10–38% of isolates) also occurred. However, only *erm*(C) was detected in the LA-MRSA ST5 isolates tested in this study. Finally, the prevalence of *fexA* in LA-MRSA ST5 was similar to that reported among LA-MRSA ST398 isolates, where 2–13% of isolates harbored *fexA* (Argudin et al., 2011; Peeters et al., 2015); however, these studies also identified the multidrug resistance gene *cfr* in 1–3% of ST398 isolates screened (Argudin et al., 2011; Peeters et al., 2015; Mutters et al., 2016), but *cfr* was not found in the LA-MRSA ST5 isolates tested here.

While phenotypic AMR prevalence among the LA-MRSA ST5 isolates evaluated here was similar to reports among LA-MRSA ST398, we identified differences in the repertoire of AMR genes harbored by ST5 isolates compared to ST398 isolates. This difference in the range or set of AMR genes harbored by LA-MRSA ST398 compared to LA-MRSA ST5 isolates could reflect specific study populations or may represent lineage specific adaptions. Lineage specific adaptions have been previously identified for disinfectants in HA-MRSA isolates and zinc resistance in LA-MRSA isolates (Hau et al., 2017g). In the present study, LA-MRSA ST5 isolates harbored *tet*(L) and *tet*(T) on an extrachromosomal plasmid. In contrast, the *tet*(M) gene is integrated into the chromosome of LA-MRSA ST398 isolates is widespread in isolates from livestock (Nesin et al., 1990; Argudin et al., 2011; Price et al., 2012; Peeters et al., 2015; Mutters et al., 2016). This may be an adaption specific to the ST398 lineage, while ST5 isolates harbor plasmid encoded tetracycline resistance. Differences in resistance genes may also be attributed to differences in the study populations. The majority of studies investigating LA-MRSA ST398 have evaluated European isolates, which are geographically distinct and may be under different selection pressures than isolates in the United States. We observed substantial between-farm and within-farm variation in both phenotypic resistance and AMR genes (**Supplementary Table S1**). Variation between farms may be associated with differences in on-farm selection pressures, in the case of MGEs encoding resistance, or the genetic background of isolates prior to introduction, such as those isolates with *parC* and *gyrA* mutations. Within farm variation of resistance phenotypes and resistance elements was also seen in LA-MRSA ST5 isolates (**Supplementary Table S1**). For example, ISU839 expressed phenotypic resistance to tetracycline class antibiotics, clindamycin, and streptomycin, while ISU837 and ISU842 were isolated from the same farm and susceptible to these antibiotics. This is due to ISU839 harboring the insertion sequence IS*Ssu5*, originally discovered in *Streptococcus suis*, that encodes *lnu*(B) and *aadE* (Chen et al., 2007), which was absent in other isolates from the same farm. Similar within farm variation has been seen in LA-MRSA ST398 isolates (Morcillo et al., 2015). Overall, there is evidence indicating LA-MRSA ST5 isolates are able to gain and lose MGEs encoding AMR genes and support concerns regarding the capacity of these isolates to disseminate AMR beyond the agricultural setting.

To begin addressing the public health concerns over the potential for LA-MRSA isolates to potentially disseminate AMR beyond the agricultural setting, we specifically selected clinical isolates from geographically distinct populations in urban environments to ensure the clinical isolates were from humans with no swine contact. We found swine associated LA-MRSA ST5 isolates exhibited resistance to fewer antibiotics than clinical MRSA ST5 isolates. More importantly, we identified separate and distinct genetic determinants of AMR harbored by clinical ST5 isolates and swine associated ST5 isolates. Collectively, our data provide a starting point for the evaluation of the swine reservoir in the United States as a potential source of AMR determinants in HA-MRSA isolates from people with no contact to swine. To fully evaluate the contribution of LA-MRSA ST5 isolates to the risk of human MRSA infections and AMR in human MRSA isolates, follow-up studies that include clinical isolates from humans with no known swine contact obtained from regions of swine production are warranted.

## Author Contributions

SH, JH, PD, TF, and TN conceived and designed the experiments, analyzed the data, contributed reagents, materials, and analysis tools, and wrote the paper. SH performed the experiments. All authors gave approval of the final version to be published and agreed to be accountable for all aspects of the work.

## Conflict of Interest Statement

The authors acknowledge receiving funding from the National Pork Board, however, declare that the funding sources in no way impacted experimental design, data analysis, manuscript preparation, or the decision to publish.

## Footnotes

1 http://mira-assembler.sourceforge.net/

2 http://www.genomicepidemiology.org/

3 https://card.mcmaster.ca/home

4 https://blast.ncbi.nlm.nih.gov/Blast.cgi

## Supplementary Material

FIGURE S1 LA-MRSA ST5 tetracycline resistance plasmid. The tetracycline resistance genes *tet*(T) (magenta) and *tet*(L) (coral) were harbored on a 9269 bp multidrug resistance plasmid that also harbored the aminoglycoside resistance gene *aadD* (light blue). This plasmid was found in 62/82 (75.6%) of all swine associated LA-MRSA ST5 isolates and had a 95.4% (62/65) correlation with tetracycline resistance.

FIGURE S2 LA-MRSA ST5 plasmid containing *erm*(C). MLS resistance in LA-MRSA ST5 was predominantly mediated by *erm*(C) (orange) in 92.3% (36/39) of isolates. This gene was found most commonly on a 2432 bp plasmid (35/36, 97.2%). Isolates carrying this plasmid were resistant to erythromycin, tilmicosin, and clindamycin.

FIGURE S3 Small plasmid carrying *aadD* found in clinical MRSA ST5 isolates. The *aadD* gene (light blue) was harbored on a 5370 bp plasmid in the majority of clinical MRSA ST5 (60/62, 96.8%). The plasmid also harbored a bleomycin resistance protein (white), which confers resistance to belomycin a glycopeptide antibiotic used for chemotherapeutic treatment of cancer.

FIGURE S4 Transposon harboring *aac(6′)-aph(2″)*. The gentamicin resistance gene *aac(6′)-aph(2″)* was harbored on the same transposon in both LA-MRSA ST5 isolates (14/82, 17.1%) and clinical MRSA ST5 isolates (9/71, 12.7%). The aminoglycoside resistance gene *aph(2″)-Ih* was found in LA-MRSA ST5 (2/82, 2.4%) and clinical MRSA ST5 (3/71, 4.2%). This gene was determined to be a truncated *aac(6′)-aph(2″)* due to its location at the end of a contig. This transposon was highly correlated with the presence of gentamicin resistance and was found in 72.7% (16/22) of gentamicin resistant LA-MRSA ST5 isolates and 100% (12/12) of clinical MRSA ST5 isolates that were phenotypically resistant to gentamicin.

TABLE S1 Source, typing, and AMR composition for all isolates used in study.
