# Supplementary figures and images for: Antimicrobial Resistance Distribution Differs Among Methicillin Resistant Staphylococcus aureus Sequence Type (ST) 5 Isolates From Health Care and Agricultural Sources
Source: Front Microbiol. 2018 Sep 11;9:2102. doi: 10.3389/fmicb.2018.02102 (PMC6143795; doi:10.3389/fmicb.2018.02102)

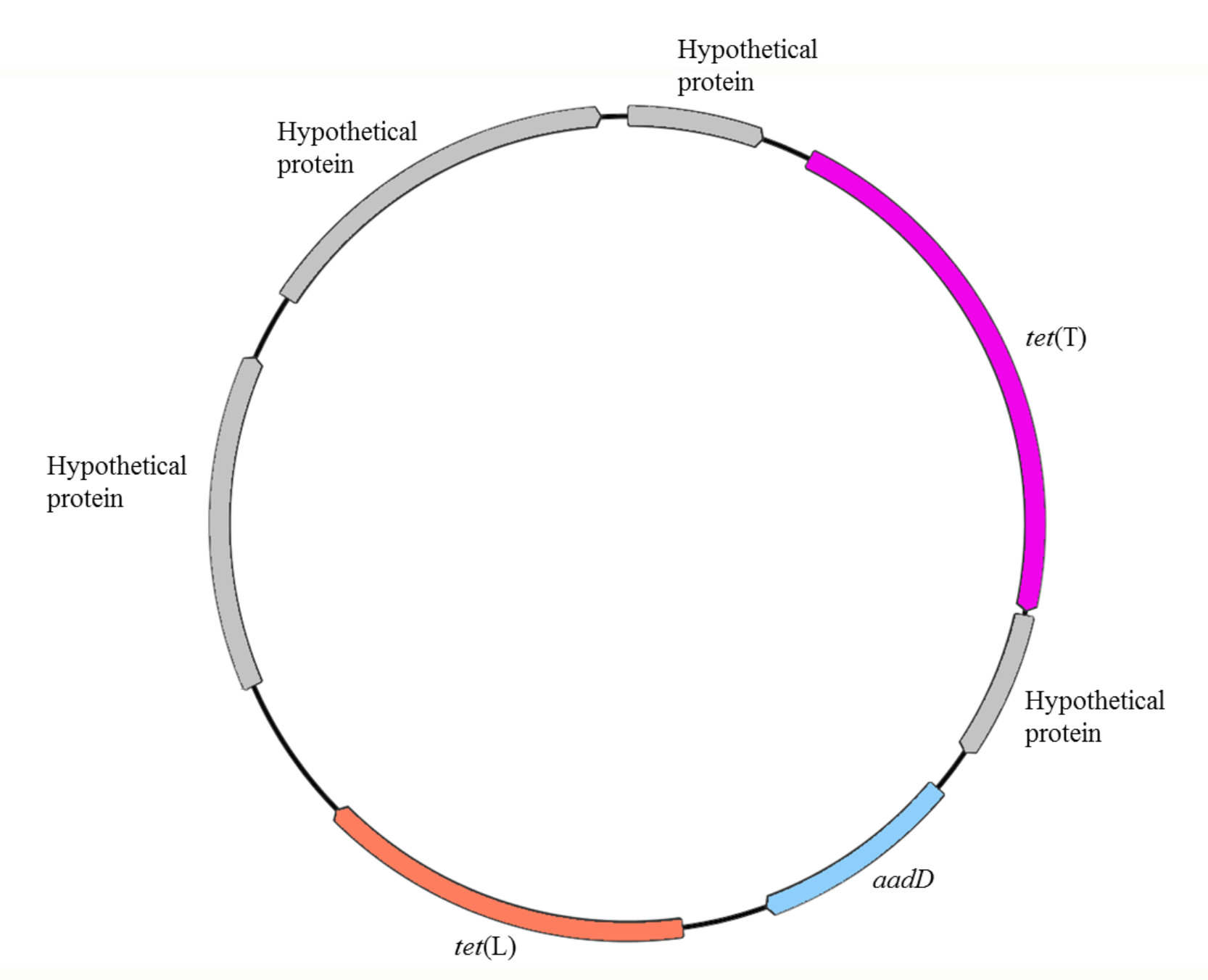

Supplement: FIGURE S1 — LA-MRSA ST5 tetracycline resistance plasmid. The tetracycline resistance genes tet(T) (magenta) and tet(L) (coral) were harbored on a 9269 bp multidrug resistance plasmid that also harbored the aminoglycoside resistance gene aadD (light blue). This plasmid was found in 62/82 (75.6%) of all swine associated LA-MRSA ST5 isolates and had a 95.4% (62/65) correlation with tetracycline resistance. [file Image_1.TIF]

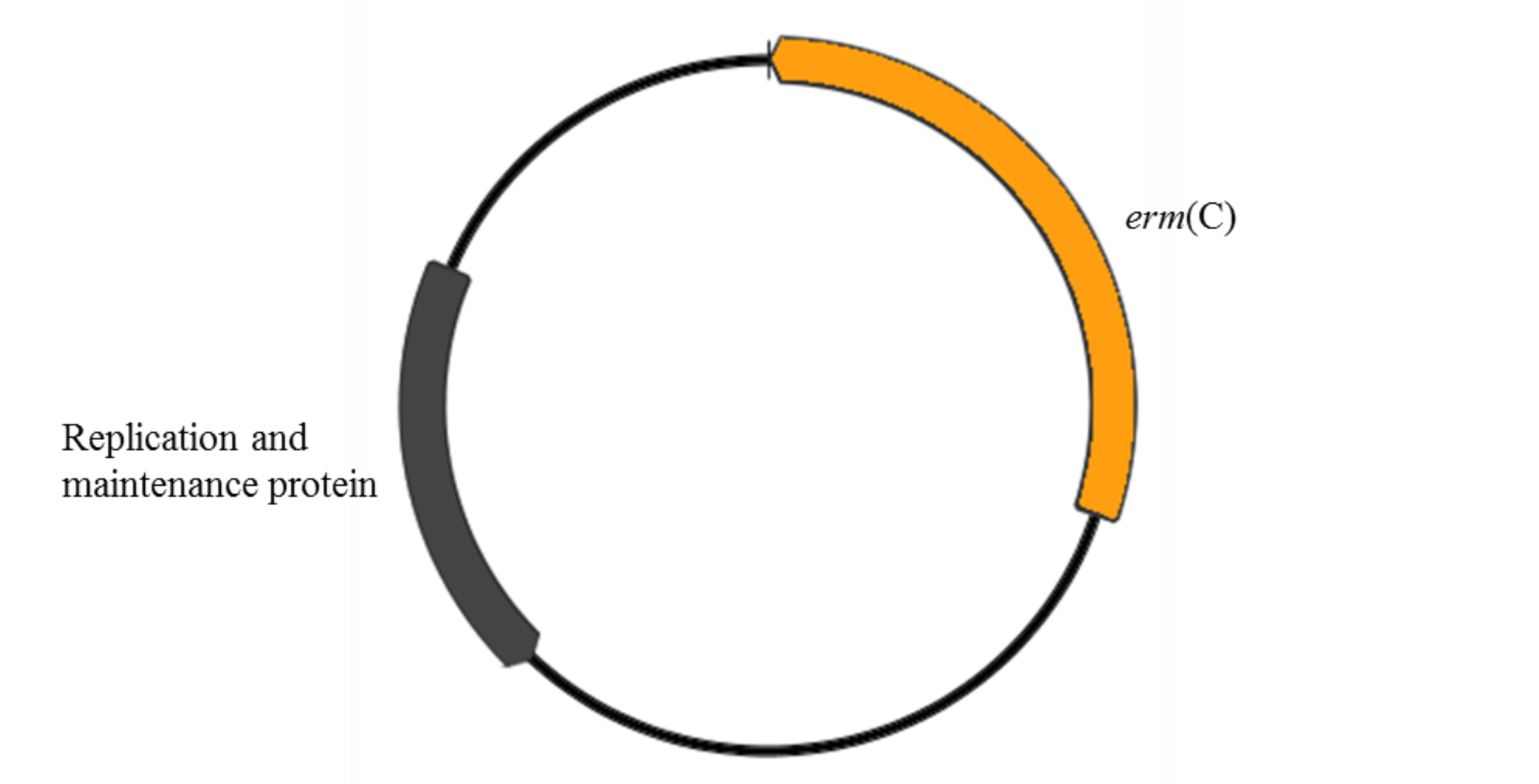

Supplement: FIGURE S2 — LA-MRSA ST5 plasmid containing erm(C). MLS resistance in LA-MRSA ST5 was predominantly mediated by erm(C) (orange) in 92.3% (36/39) of isolates. This gene was found most commonly on a 2432 bp plasmid (35/36, 97.2%). Isolates carrying this plasmid were resistant to erythromycin, tilmicosin, and clindamycin. [file Image_2.TIF]

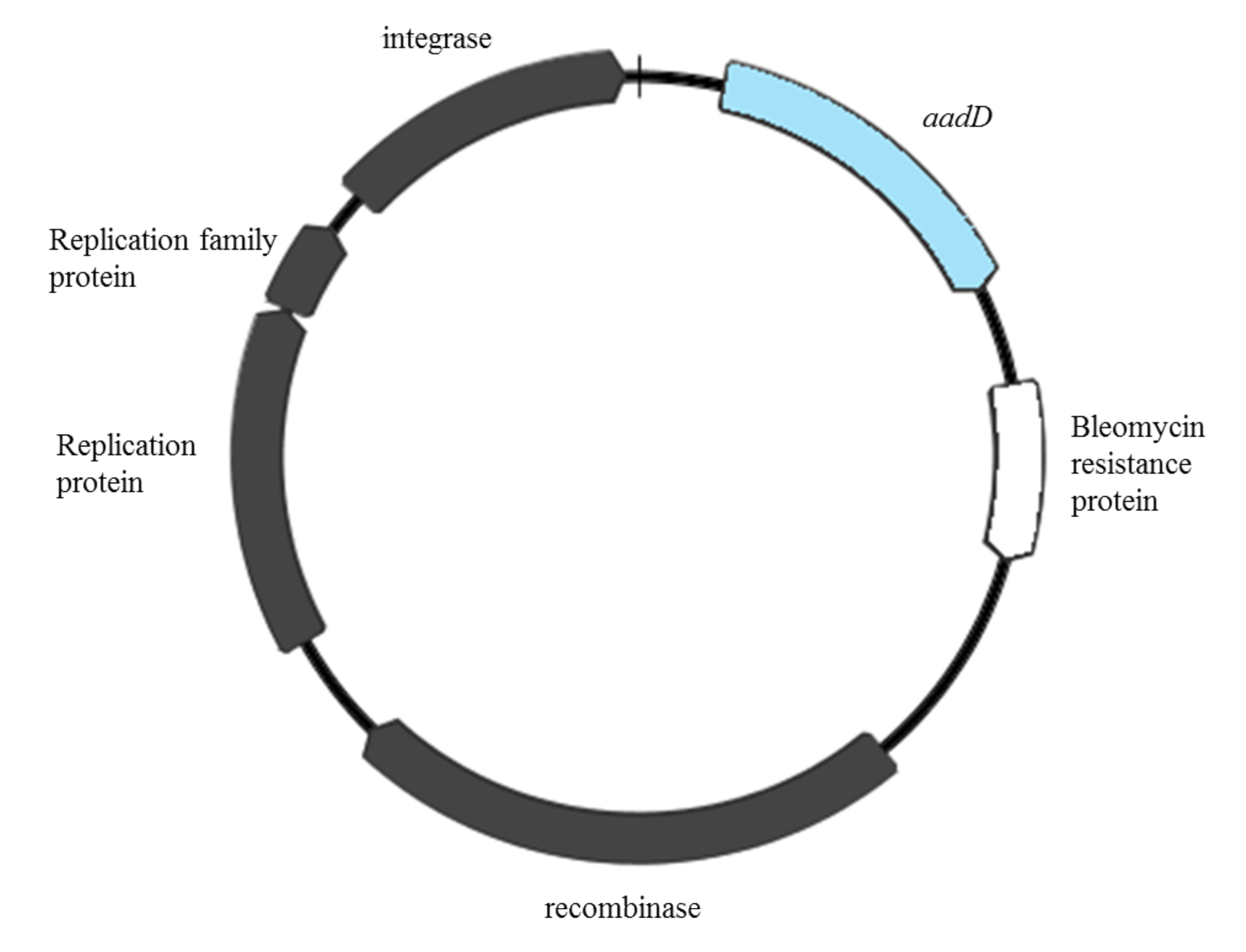

Supplement: FIGURE S3 — Small plasmid carrying aadD found in clinical MRSA ST5 isolates. The aadD gene (light blue) was harbored on a 5370 bp plasmid in the majority of clinical MRSA ST5 (60/62, 96.8%). The plasmid also harbored a bleomycin resistance protein (white), which confers resistance to belomycin a glycopeptide antibiotic used for chemotherapeutic treatment of cancer. [file Image_3.TIF]

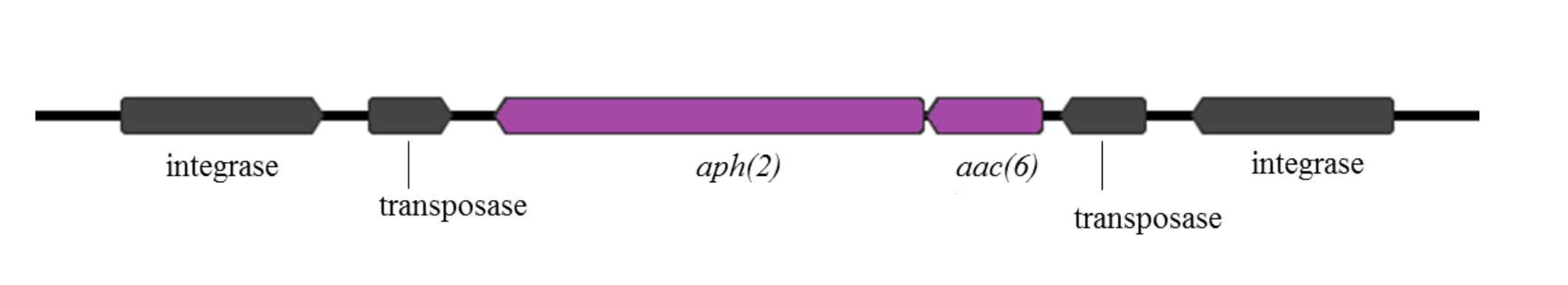

Supplement: FIGURE S4 — Transposon harboring aac(6′)-aph(2″). The gentamicin resistance gene aac(6′)-aph(2″) was harbored on the same transposon in both LA-MRSA ST5 isolates (14/82, 17.1%) and clinical MRSA ST5 isolates (9/71, 12.7%). The aminoglycoside resistance gene aph(2″)-Ih was found in LA-MRSA ST5 (2/82, 2.4%) and clinical MRSA ST5 (3/71, 4.2%). This gene was determined to be a truncated aac(6′)-aph(2″) due to its location at the end of a contig. This transposon was highly correlated with the presence of gentamicin resistance and was found in 72.7% (16/22) of gentamicin resistant LA-MRSA ST5 isolates and 100% (12/12) of clinical MRSA ST5 isolates that were phenotypically resistant to gentamicin. [file Image_4.TIF]
